# Supplementary material for: Simulating groundstate and dynamical quantum phase transitions on a superconducting quantum computer
Source: Nat Commun. 2022 Oct 10;13:5977. doi: 10.1038/s41467-022-33737-4 (PMC9550817; doi:10.1038/s41467-022-33737-4)
Supplement: Supplementary file 1 — Supplementary Information [file 41467_2022_33737_MOESM1_ESM.pdf]

Supplementary Information: Simulating groundstate and dynamical quantum phase transitions on a superconducting quantum computer

James Dborin[1], Vinul Wimalaweera[1], F. Barratt[2], Eric Ostby[3], Thomas E. O'Brien[3], and A. G. Green*[1]

[1]London Centre for Nanotechnology, University College London, Gordon St., London, WC1H 0AH, United Kingdom
[2]Department of Physics, University of Massachusetts, Amherst, MA 01003, USA
[3]Google Quantum AI, 80636 Munich, Germany

September 28, 2022

Abstract

In this supplementary material we further explain the methods used to construct circuits for time-evolution. We pay particular attention to the tradeoffs between circuit approximations and fidelity in constructing cost-functions.

1 Supplementary Note 1 - Order of Trotterisation

One of the key refinement parameters in time-evolving circuit states is the order of Trotterisation. Supplementary Fig.1 illustrates the different transfer matrices that result from a second-order Trotterisation and from a first-order Trotterisation that we have simplified using a property of translationally invariant states. Because of the projection back to translationally invariant states, this update is in fact effective to higher order in dt. This can be demonstrated as follows: the time-dependent variational principle equations for evolving a translationally invariant state with just the even- or odd-bond parts of the Hamiltonian are identical to evolving using the full Hamiltonian divided by two[1]. Our algorithms are equivalent to a discretisation of the time-dependent variational principle[2], since the latter continuously projects the evolved state back to the state on the MPS manifold that optimises fidelity and our algorithms involve an explicit optimisation of fidelity at discrete time-steps. The results presented below and in the main paper are exclusively for this simplified first-order Trotterisation in order to avoid the deeper circuit required for higher-order Trotterisation.

2 Supplementary Note 2 - Cost Functions for Time-evolution

As discussed in Section II C of the main text, finding an appropriate cost-function for time-evolution quantum circuit iMPS involves a subtle trade-off between the analytical approximation to the eigenvalue of the transfer matrix - determined by order of Trotterisation, order of power method used, and accuracy of approximations to the transfer matrix fixed-points - and the infidelities of representing this circuit on a real device. Here we give results for a number of different cost functions, the dynamics that they predict in the absence of circuit noise and their realisation on the Rainbow device.

*email: andrew.green@ucl.ac.uk

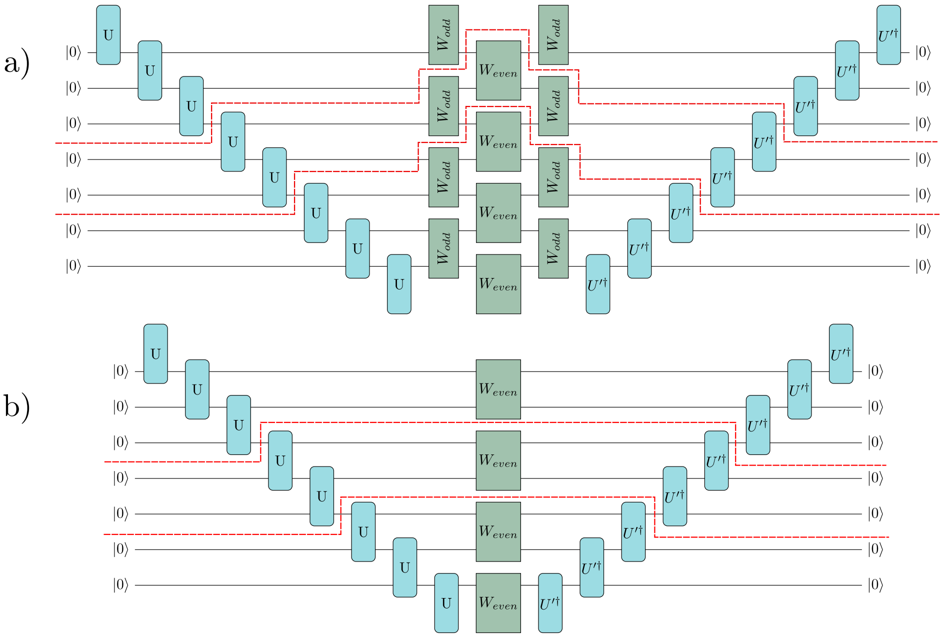

Supplementary Figure 1: **Time-evolution Transfer Matrix** a) The overlap $|\langle\psi(U')|e^{\mathcal{H}dt}|\psi(U)\rangle|^2$, calculated with a second order Trotterisation. Here $W_{even} = e^{i\mathcal{H}_e dt}$ and $W_{odd} = e^{i\mathcal{H}_o dt/2}$. This overlap is formally an infinite width and depth circuit. b) An expression for the overlap with one half timestep as used here. Mapping back to a translationally-invarient variational manifold increase sthe effective order of the time-integration.

2.1 Approximating the right fixed point with $|0\rangle\langle 0|$

First we show in Figs. 2 and 3 the result of using an identity approximation to the left eigenvalue and a simple $|0\rangle\langle 0|$ approximation to the right eigenvalue. As shown in 2, this performs very poorly when using the ratio C_2/C_1 to estimate the principle eigenvalue of the transfer matrix. This is true even when the circuits are calculated in simulations in the absence of noise. This is apparently due to the low order of the power method, despite the good approximation to the left fixed point. When the same method is used to calculate C_5/C_4 the approximation performs well in simulations without noise.

Curiously, simply using C_2 as an approximation to λ^2 performs much better. Supplementary Fig.3 shows that, in the absence of circuit errors, this circuit does a passable job of capturing the dynamical quantum phase transition in the quench dynamics of the quantum Ising model. However, when implemented on the Rainbow device, the optimum measured cost function is not at the correct updated values - even after we carry out our rescaling to account for depolarisation error.

2.2 Improved approximations to the right fixed-point.

While in principle, a good approximation to the principal eigenvalue of the transfer matrix can be found using a good initial approximation to only either the left or right fixed-points of the transfer matrix. We find in practice that we get much better results if our approximations to both left and right fixed points are good. Supplementary Figs.4 and **??** show the results of progressive improvements to the approximation.

In Supplementary Fig. 4 we show the results of using the same circuit as in Fig. 3 in the main paper, but measuring the probability of $|0\rangle^{\otimes 6}$ at the output rather than post-selecting the two top right qubits on $|0\rangle$. This amounts to using U and U' to construct an approximation to the right fixed-point. The resulting circuit does a passable job of capturing the dynamical quantum phase transition in the absence of circuit errors, and the cost function is apparently tracked rather well when implemented on the Rainbow device. However, the measured optimum value of this cost function is not correct. Post-selecting on the top right qubits corrects these deficiencies. This procedure factors out the U'-dependent norm of the approximation

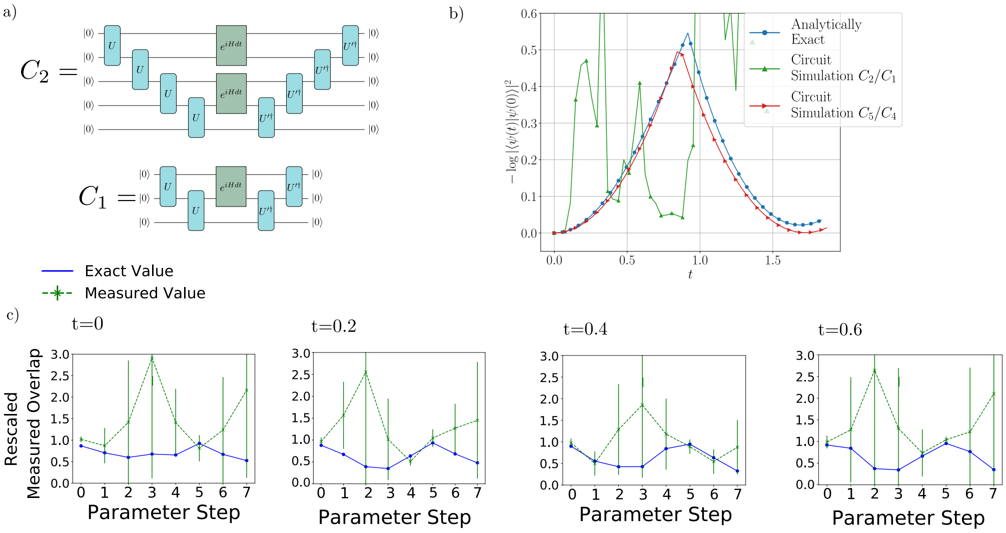

Supplementary Figure 2: **Time-Evolution Circuits and Results: approximating the right fixed point with $|0\rangle\langle 0|$ Ratio of $C_2/C_1 \approx \lambda$.** a) *Time-evolution circuit:* This circuit approximates the left fixed point as $\boldsymbol{I}$ and the right fixed point as $|0\rangle\langle 0|$. The probability of measuring $|0\rangle^{\otimes N}$ at the output is divided by the appropriate Loschmidt echo and the ratio of the output with two copies of the transfer matrix divided by the output for a circuit with one copy of the transfer matrix. This gives an approximation to λ — the principal eigenvalue of the transfer matrix. b) *Dynamical Quantum Phase Transition in the Quantum Ising Model:* Exact analytical results of the time evolution and a simulation using direct stochastic optimisation of the circuit in a) in the absence of noise. Taking the ratio of C_2/C_1 approximated with the circuit shown in a) does not reproduce the correct time evolution. Calculating C_5/C_4 using the same approximation for the right fixed point does a much better job at capturing the time-evolution suggesting that it is the low order of the power method that leads to the failure. c) *Cost-function evaluated on Google's Rainbow Device:* The cost function evaluated along a linear interpolation from $U' = U$ through the optimal value of U' as in Fig.3 of the main text. Though this displays a peak at the optimum value it is not the global optimum of the circuit.

to the right fixed-point, giving better performance both under simulation in the absence of error and when implemented on the Rainbow device.

Finally, we report on two further approximations to the right fixed point that do not require post-selection, and may perform even better than the circuit reported in the main paper. Unfortunately, it was not possible to test these circuits on the Rainbow device as they were discovered after the machine was taken offline. These correspond to approximating the right fixed-point by that obtained when overlapping the state described by U with itself. This is a good approximation since evolving the state described by U' backwards in time ought to recover the state described by U. The two approximation shown in Supplementary Fig.**??** correspond to using the fixed point determined by solving the fixed-point equations of Fig.4c in the main paper for $V(U)$ and an approximation to this obtained by simply overlapping U with itself to construct an approximation to the right fixed point. Both approximations do an excellent job of capturing the evolution in the absence of noise and we believe will provide a good starting point for future investigation.

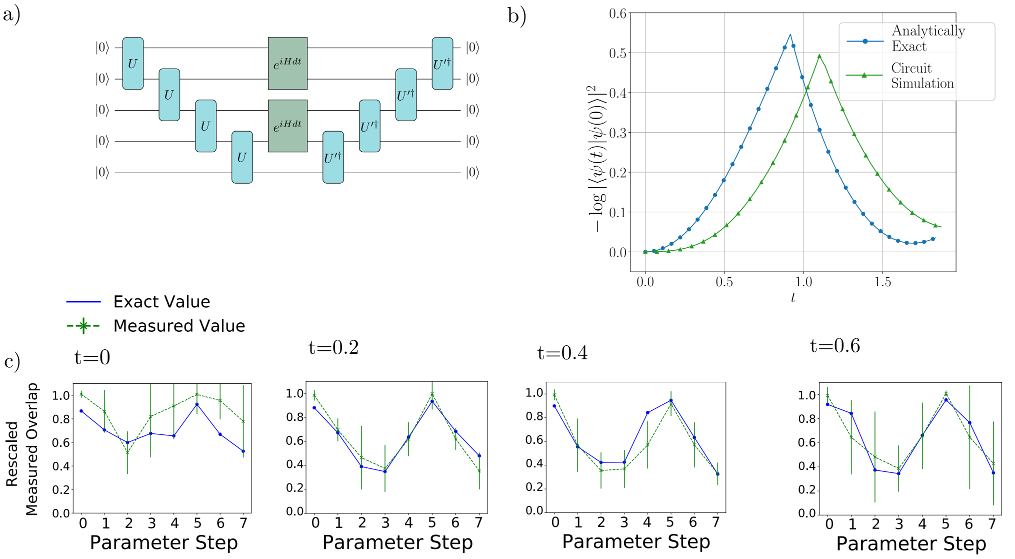

Supplementary Figure 3: **Time-Evolution Circuits and Results: approximating the right fixed point with $|0\rangle\langle 0|\ C_2 \approx \lambda^2$.** a) *Time-evolution circuit:* Here we use the first of the two circuits used in Supplementary Fig. 2. The probability of measuring $|0\rangle^{\otimes N}$ at the output is divided by the appropriate Loschmidt echo. This gives an approximation to λ^2 — the square of the principal eigenvalue of the transfer matrix. b) *Dynamical Quantum Phase Transition in the Quantum Ising Model:* Exact analytical results of the time evolution and from a simulation using direct stochastic optimisation of the circuit in a) in the absence of noise. Surprisingly, although the same circuit is used as in Supplementary Fig. 1, in this case, the circuit captures key features of the evolution when calculated in the absence of noise. c) *Cost-function evaluated on Google's Rainbow Device:* The cost function evaluated along a linear interpolation between the initial and optimal parameters as in Fig.3 of the main text. Although the cost function measured on the Rainbow deice does track its calculation in the absence of noise, we find that the peak does not always occur at the optimal value.

Supplementary References

[1] Haegeman, J. *et al.* Time-dependent variational principle for quantum lattices. *Phys. Rev. Lett.* **107**, 070601 (2011). URL `https://link.aps.org/doi/10.1103/PhysRevLett.107.070601`.

[2] Barratt, F. *et al.* Parallel quantum simulation of large systems on small nisq computers. *npj Quantum Information* **7**, 1–7 (2021).
